# The impact of vitamin D supplementation on peripheral neuropathy in a sample of Egyptian prediabetic individuals

**DOI:** 10.12688/f1000research.55221.1

**Published:** 2021-08-16

**Authors:** Mohamed Reda Halawa, Iman Zaky Ahmed, Nahla Fawzy Abouelezz, Nagwa Roushdy Mohamed, Naira Hany Abdelaziz Khalil, Laila Mahmoud Ali Hendawy

**Affiliations:** 1 Internal Medicine and Endocrinology Department, Ain Shams University, Cairo, 11566, Egypt; 2 Community, Environmental and Occupational Medicine Department, Ain Shams University, Cairo, 1181, Egypt

**Keywords:** prediabetes, vitamin D, peripheral neuropathy, neuropathic score

## Abstract

**Background:** Vitamin D deficiency is seen more frequently in diabetic patients with distal symmetrical polyneuropathy **.** Unfortunately, there is a shortage of data concerning prediabetic individuals with peripheral neuropathy (PN). Therefore, we aimed to study the association of vitamin D deficiency with PN severity and to determine the effect of vitamin D supplementation on PN in prediabetics.

**Methods** **: **A case-control study was conducted consisting of 178 prediabetic individuals recruited from the outpatient department of the National Institute of Diabetes and Endocrinology, Cairo, Egypt. All patients were screened for PN using clinical examination and Douleur Neuropathique 4 diagnostic questionnaire (DN4). They were divided into 89 patients with and 89 patients without PN (group A and B). Group A was assessed for neuropathic severity using the Short-Form McGill Pain Questionnaire (SF-MPQ). In addition, 25-hydroxyvitamin D, ionized calcium, phosphorus, parathyroid hormone (PTH), glycated hemoglobin (HbA1c), fasting blood glucose (FBG), 2-hour post 75g glucose (2h-75g glucose) and lipid profile were measured. Prediabetic patients with PN were given vitamin D3 200.000 IU IM monthly for three months. After three months, clinical assessment, DN4, SF-MPQ and all laboratory measures were repeated.

**Results:** Vitamin D level was negatively correlated with neuropathy score and severity (r = -0.65, -0.47, p <0.001) among group A. Moreover, vitamin D level was an independent predictor of neuropathic severity (odds ratio -0.18, 95% CI -0.33 -0.03, P ≤ 0.05). Supplementation of vitamin D resulted in a highly significant improvement in glycemic parameters and lipid profile, p ≤ 0.001. Interestingly, neuropathy score and severity before vitamin D supplementation were (6.4 ± 1.6 and 28.3 ± 7.2) and after became (2.5 ± 0.9 and 17 ± 6.3, p ≤ 0.001).

**Conclusion:** Vitamin D deficiency is an independent risk factor for PN. Correction of vitamin D deficiency improves glycemic parameters, PN score and severity.

Editorial note: **27th August 2021:** Since publication of this article, the F1000Research editorial team have been made aware of some potential issues and inconsistencies in the data and analysis. As a member of the Committee on Publication Ethics (COPE), F1000Research provides an ethical publishing framework in accordance with COPE's codes of conduct for editors and publishers. We are investigating these issues with the authors of the manuscript. This Editorial Note has been posted to inform readers of a potential, not yet resolved, problem with this article, and will remain in place whilst investigations are ongoing. Any further action will be dependent on the outcome of these discussions and peer review activity has been suspended as a precaution in the meantime.

## Introduction

Diabetes mellitus (DM), a significant world health problem, is a metabolic disease, which occurs due to a defect in insulin release and or insulin resistance ^1^. Globally, the prevalence of type 2 diabetes (T2DM) is high and rising across all regions ^2^.

There is a higher frequency of idiopathic polyneuropathy, small fiber neuropathy and painful sensory neuropathy among prediabetics. These findings suggest an involvement of the small unmyelinated nerve fibers that carry pain, temperature, and regulate autonomic function during prediabetes, before the development of diabetes ^3^.

Vitamin D, which is a fat-soluble hormone, has multiple physiological roles, which extends far beyond calcium metabolism ^4^. Vitamin D deficiency is a worldwide health problem, patients with prediabetes, T2DM, gestational diabetes and obesity represent a high-risk group ^5^.

Recently, a lot of studies have been done to assess the association between vitamin D level and the diabetic peripheral neuropathy in patients with diabetes mellitus and to study the effect of vitamin D on painful neuropathy, but there is a lack of data concerning prediabetic individuals ^1^. 

The aim of this work was to assess the association of vitamin D deficiency with peripheral neuropathy severity and determine the effect of vitamin D supplementation on peripheral neuropathy in prediabetics.

## Methods

An interventional case-control study was conducted on 178 prediabetic individuals aged 18–60 years diagnosed, according to the American Diabetes Association 2019, with impaired fasting (100–125 mg/dl) and/or impaired glucose tolerance (140–199 mg/dl), and/or glycated haemoglobin (5.7–6.4%) ^6^. Participants were recruited from the National Institute of Diabetes and Endocrinology (NIDE), Cairo, Egypt, in the period from September 2018 to March 2019 after proven informed written consent. Ethical approval of the study was obtained from the Local Research Ethical Committee (REC) of the Faculty of Medicine, Ain Shams University. FWA 000017858. 

All participants were subjected to full medical history including smoking habits, alcohol consumption, drug history, thorough clinical examination including blood pressure, weight, height and BMI.

### Screening for peripheral neuropathy

All participants were screened for peripheral neuropathy by 10 g monofilament for assessing the loss of protective sensation, tuning fork (vibration sense testing using a 128-Hz tuning fork), ankle reflex, pinprick (for perception of pain) and Douleur Neuropathic 4 diagnostic questionnaire (DN4) ‎ ^7^ that assesses symptoms reflecting pain in the form of burning, painful, cold, electric shocks, tingling, pins and needles. If the patients score is ≥4 the patient likely suffers from neuropathic pain. Patients found to have peripheral neuropathy were given the Short-Form McGill Pain Questionnaire (SF-MPQ) ^8^ that assesses the severity of pain; an increase in the score indicates increasing severity.

The 178 prediabetic individuals were divided into two groups (Group A) 89 with peripheral neuropathy & (Group B) 89 without peripheral neuropathy. Patients of group A were given vitamin D (cholecalciferol) (200.000 IU) intramuscular every month for three successive months. These clinical assessments were repeated in the last visit after three months to assess the improvement in peripheral neuropathy in those patients. Retesting is advised after three months, as suppression of parathyroid hormone after supplementation with cholecalciferol takes at least three months and the response differs between individuals. So, most guidelines recommend repeat testing after three months ^9^.

### Laboratory studies

•  Subjects were first instructed to fast for eight hours (overnight fasting), 10 ml of venous blood were then collected by venipuncture without tourniquet.

•  2 ml of the collected blood were taken in an EDTA containing tube for the assay of the glycated hemoglobin and it was stored at 4°C to be carried out within one week.

•  2 ml were taken in a fluoride containing tube and then separated by centrifugation and the sample was used for measurement of TSH, serum Ca, phosphorus, serum creatinine, PTH and 25(OH)vitamin D.

•  2 ml sample were collected two hours after 75 g oral glucose load for the measurement of the 2h-OGTT.

•  On a separate day, 2 ml of venous blood were collected by venipuncture (after an overnight 12 hour fast), the sample was collected in a fluoride containing tube and then separated by centrifugation and used for measurement of total lipid profile (total cholesterol, low density lipoprotein (LDL), triglycerides (TG)) by enzyme colorimetric assay.

▪ Total cholesterol level was measured by Quantitative Enzymatic-Colorimetric assay (Catalogue Number: 1010/ manufacturer: Stanbio-Laboratory,Inc., USA/ Boerne, Texas/ 1/2018)
▪ Triglyceride level was measured by Quantitative Enzymatic-Colorimetric assay (Stanbio LiquiColor Triglycerides/ Catalog Number: 2100/ manufacturer: Stanbio-Laboratory,Inc., USA/ Boerne, Texas, USA/ 03/2018)
▪ LDL cholesterol can be determined as the difference between total cholesterol and the cholesterol content of the supernatant (HDL and VLDL) after precipitation of LDL fraction by polyvinyl sulphate in the presence of polyethylene-glycol monomethyl ether. Calculation LDL= Cholesterol- (HDL+ Triglyceride/5)
▪ HDL level was measured by Quantitative Enzymatic-Colorimetric assay (Stanbio HDL cholesterol/ Catalog Number: 0599/ manufacturer: Stanbio-Laboratory,Inc., USA/ Boerne, Texas, USA/ 02/2018)
▪ Serum 25- hydroxyvitamin D level was measured by an ELISA kit, which is a solid phase enzyme-linked immunosorbent assay (ELISA, Catalogue Number: 10501, Chemux Bioscience, Inc., Hayward, CA/ 10/2018).
▪ Parathormone level was measured by an ELISA kit with a normal range of 10–55 pg/ml (ELISA, Catalogue Number: KAP1481, DIAsource ImmunoAssays S.A, Nivelle, Belgium/ 2/2018).
▪ Glycated hemoglobin was measured by quantitative colorimetric determination of glycated haemoglobin in whole blood (Catalog Number: 0350/ manufacturer: Stanbio-Laboratory, Inc., Boerne, Texas, USA/ 06/2018).
▪ Fasting blood glucose, 75-oral glucose tolerance test (2h-OGTT) were measured by Stanbio Glucose LiquiColor (Oxidase) (Catalog Number: 1070, manufacturer: Stanbio-Laboratory,Inc., USA, Boerne, Texas, USA/ 04/2018).
▪ All laboratory tests were conducted at the beginning of the study and after three months of supplementation with vitamin D.
▪ Vitamin D status was assessed according to Hovsepian *et al*.,
   Sufficiency >30ng/ml, Insufficiency (20–29) ng/ml, Deficiency <20 ng/ml ^10^

### Exclusion criteria

Patients with renal impairment, hypo or hyperthyroidism, patients on vitamin D supplementation or antiepileptic or any medication affecting calcium and vitamin D level, pregnant or breast-feeding females were excluded from the study. 

### Statistical and study design:

A sample size of 175 cases of prediabetics was calculated using Epi Info ^™^7 program using prevalence of vitamin D deficiency among prediabetics = (87 ± 5) % with accepted range (82–92) % at 95% C.I. ^11^

### Statistical analysis

The data were analyzed using SPSS version 17 (IBM Corporation, USA) (RRID:SCR_019096) (An open-access alternative that can perform an equivalent function is the R Stats package) (RRID:SCR_001905). The quantitative data that were measured first were: (Age (Years), BMI (kg/m2), Systolic BP (mmHg), Diastolic BP (mmHg), HbA1c (%), 2h-75g glucose (mg/dl), S.T. cholesterol (mg/dl), S.LDL (mg/dl), S.HDL (mg/dl), S.TG (mg/dl), S. Creatinine (mg/dl), S. TSH (mU/L), 25 (OH) Vit D (ng/ml), S. Ionized Ca (mg/dl), S. Phosphorus (mg/dl), S. PTH (pg/ml) )and they were presented as mean and standard deviation and the Student’s T-test was used to compare two independent groups (group A and group B) with quantitative data. Second, (HbA1C (%), FBG (mg/dl), 2h-75g glucose (mg/dl)), were measured and they were presented as mean and standard deviation and the paired T-test was used to compare group A before and after vitamin D supplementation. Spearman correlation coefficients were used to assess the correlation between two quantitative parameters in the same group A before and after vitamin D supplementation. They were used to compare between vitamin D level with the severity of peripheral neuropathy score and with the DN4 questionnaire score. Regarding qualitative data, we measured vitamin D status (Sufficient, Insufficient, Deficient) and they were presented as numbers and percentages and the Chi-Square test was used to compare two independent groups (group A and group B) with qualitative data. Additional qualitative data that were measured were clinical examination for peripheral neuropathy using ankle reflex, tuning fork (vibration) and 10 g monofilament and they were presented as numbers and percentages and the Chi-square test was used to compared group A before and after vitamin D supplementation. The linear regression analysis test was used to identify the strength of the effect of the independent variables on a dependent variable. The confidence interval was set to 95% and the margin of error accepted was set to 5%. (P > 0.05): Non-significant (NS), (P < 0.05): Significant (S) and (P < 0.001): Highly significant (HS) ^12^.

## Results

 Comparison between the two studied groups regarding clinical and laboratory characteristics is shown in Table 1.

**Table 1.** Comparison between the two studied groups before vitamin D injection.

	Group(A) (N=89)	Group (B) (N=89)	Sig	Paired t test
	Mean±SD	Mean±SD	P-value	T-test
**Age** **(Years)**	50.416±7.9841	42.876±10.4183	**≤0.001**	-5.419
**BMI** **(kg/m2)**	30.416±2.9573	30.236±3.2193	0.699	-0.388
**Systolic BP** **(mmHg)**	131.52±10.068	129.82±10.784	0.28	-1.08
**Diastolic BP** **(mmHg)**	80.28±9.925	74.38±8.147	**≤0.001**	-4.33
**HbA1c** **(%)**	5.94±0.20	5.91±0.22	0.353	-0.931
**2h-75g glucose** **(mg/dl)**	150.04±26.18	121.75±29.92	**≤0.001**	-6.713
**S.T.cholesterol** **(mg/dl)**	197.730±25.55	191.32±30.04	0.127	-1.532
**S.LDL** **(mg/dl)**	135.00±19.76	112.48±29.37	**≤0.001**	-6.000
**S.HDL** **(mg/dl)**	43.70±12.70	55.70±15.10	**≤0.001**	5.7
**S.TG** **(mg/dl)**	112.58±27.88	115.12±21.22	0.495	0.684
**S.Creatinine** **(mg/dl)**	0.62±0.11	0.67±0.18	**≤0.05**	2.401
**S. TSH** **(mU/L)**	3.01±0.85	2.91±0.90	0.428	-0.794
**25 (OH) Vit D** **(ng/ml)**	13.95±6.36	14.59±3.93	0.423	0.804
**S. Ionized Ca** **(mg/dl)**	4.591±0.4220	4.244±0.5114	≤ **0.001**	-4.940
**S. Phosphorus** **(mg/dl)**	3.434±0.1167	3.582±0.2203	≤ **0.001**	5.612
**S. PTH** **(pg/ml)**	80.22±18.07	72.34±16.67	≤ **0.05**	-3.021

*BMI= body mass index, BP= blood pressure, HbA1c = glycated haemoglobin, 2h-75g glucose = 2 hour post 75g glucose, S.T.cholesterol = serum total cholesterol, S.LDL = serum low density lipoprotein, , S.HDL = serum high density lipoprotein, S.TG = serum triglycerides, S. TSH= thyroid stimulating hormone, 25 (OH) Vit D= 25 hydroxy Vitamin D, S. Ionized Ca= serum ionized calcium, S.PTH = serum parathyroid hormone.

Upon assessment of vitamin D status among our patients we found that 27 (15%) patients were insufficient, 151 (85%) were deficient and none were sufficient. Regarding group (A): 18 (20.2%) were insufficient, 71 (79.8%) were deficient and none were sufficient; while group (B): 9 (10.1%) were insufficient, 80 (89.9%) were deficient and none were sufficient ( Table 2); with a non-significant difference in vitamin D level between the two groups (13.957 ± 6.3603 ng/ml (group A) vs 14.594 ± 3.9318 ng/mL (group B) (P>0.05) ( Table 1). 

**Table 2.** Vitamin D status among the studied groups.

	Vitamin D status						Total		P-value
	Sufficient		Insufficient		Deficient				
	Number	%	Number	%	Number	%	Number	%	
**Group (A)**	0	0%	18	20.2	71	79.8	89	100	**≤0.001**
**Group(B)**	0	0%	9	10.1	80	89.9	89	100	
**Total**	0	0%	27	15	151	85	178	100	

*Group A= Prediabetic patients with peripheral neuropathy Group B= Prediabetic patients without peripheral neuropathy

A highly significant negative correlation was found between vitamin D level and the severity of peripheral neuropathy score (r = -0.472) (P ≤ 0.001) ( Figure 1a), as shown by the SF-MPQ. We also found a highly significant negative correlation between vitamin D level and the DN4 questionnaire score (pain score) (r = -0.647) (P ≤ 0.001) ( Figure 1b).

**Figure 1a.**
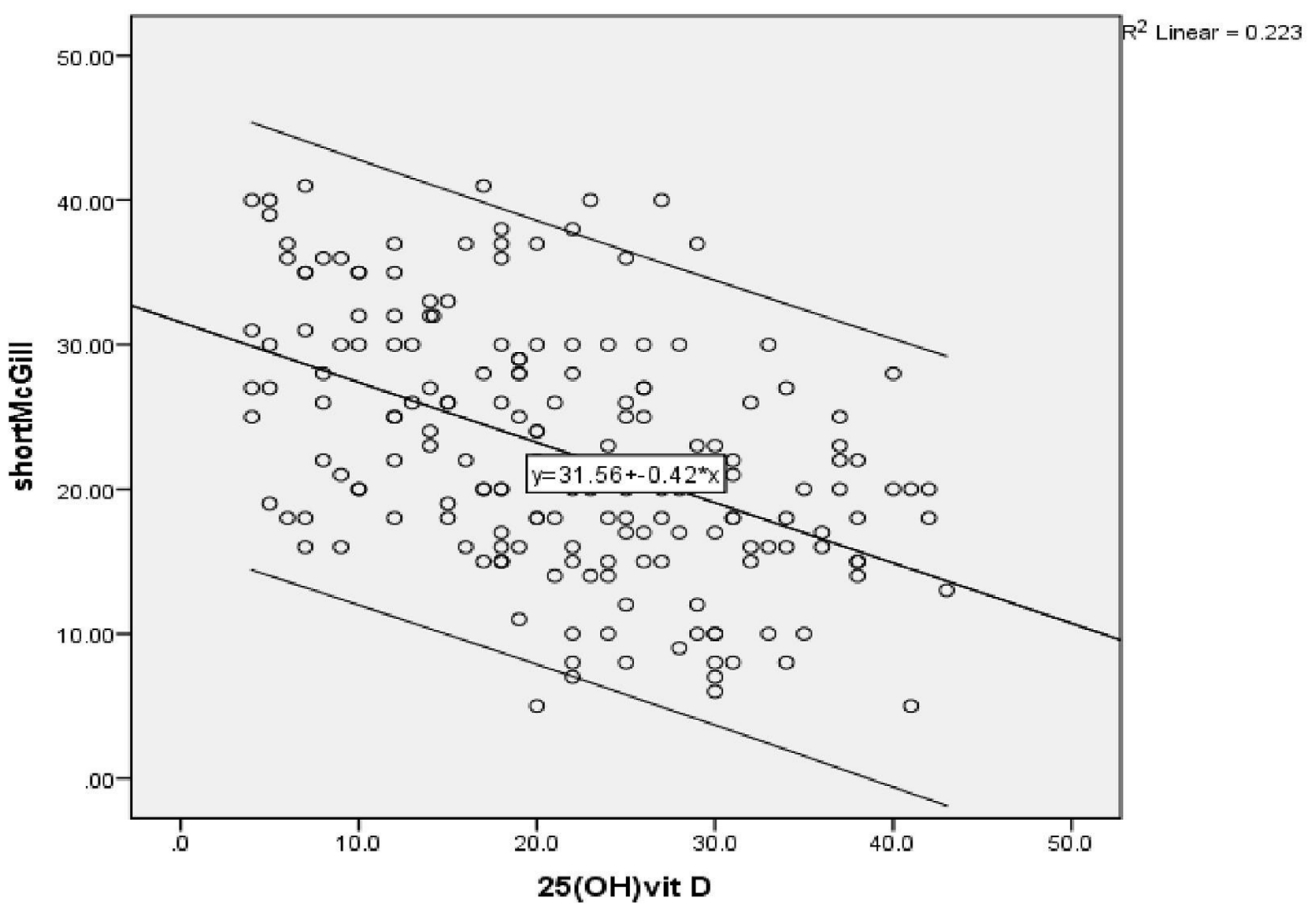
Correlation between vitamin D level and severity of peripheral neuropathy assessed by the Short-Form McGill Pain Questionnaire in group (A) before vitamin D injection.

**Figure 1b.**
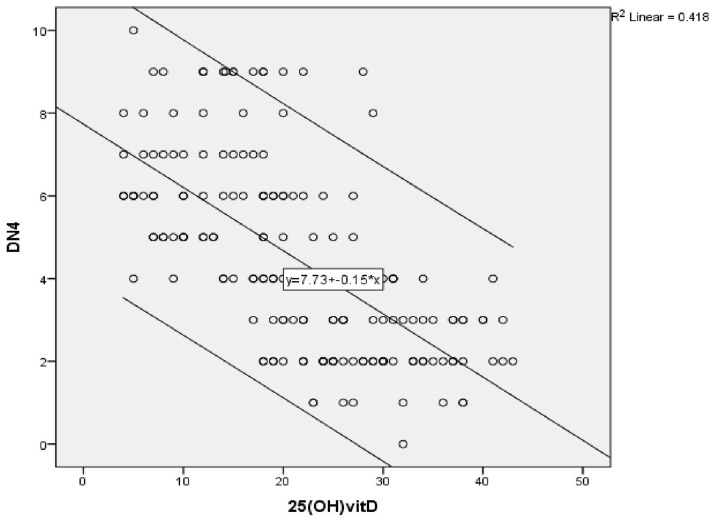
Correlation between vitamin D level and Douleur Neuropathique 4 score of neuropathic pain in group (A) before vitamin D injection.

Linear regression analysis showing correlations between the SF-MPQ and different parameters in the studied group showed that 25 (OH) vitamin D, Serum HbA1c, 2 hours PP are predictors of neuropathy severity after adjustment of age, sex, BMI and other lab parameters (OR -0.178, 95% CI -0.32– -0.03) (OR 4.846, 95% CI 0.19–9.5) (OR 0.05, 95% CI 0.005–0.104) (P≤0.05).

### After vitamin D supplementation

***Laboratory data.*** There was a highly significant improvement in vitamin D level in group (A) after intramuscular injection of vitamin D, from which 42 (47%) prediabetic patients became sufficient, 38 (42.7%) became insufficient and only 9 (10.1%) remained deficient (P≤0.001). There was a highly significant improvement of glycemic profile as shown in ( Table 3).

**Table 3.** Comparison between group (A) before and after vitamin D supplementation regarding glycemic profile.

	Group (A) before vitamin D (N=89)	Group (A) after vitamin D (N=89)	Sig.	Paired t test
	Mean±SD	Mean±SD	P-value	t-test
**HbA1C** **(%)**	5.94±0.20	5.707±0.33	≤ **0.001**	7.45
**FBG** **(mg/dl)**	101.921±15.2831	92.258±15.2722	≤ **0.001**	6.203
**2h-75g glucose** **(mg/dl)**	150.045±26.1816	102.000±16.7359	≤ **0.001**	17.309

*HbA1c = glycated haemoglobin, FBG= fasting blood glucose, 2h-75g glucose=2-hour post 75g glucose

### Neuropathic pain score and its severity

**Figure 2.**
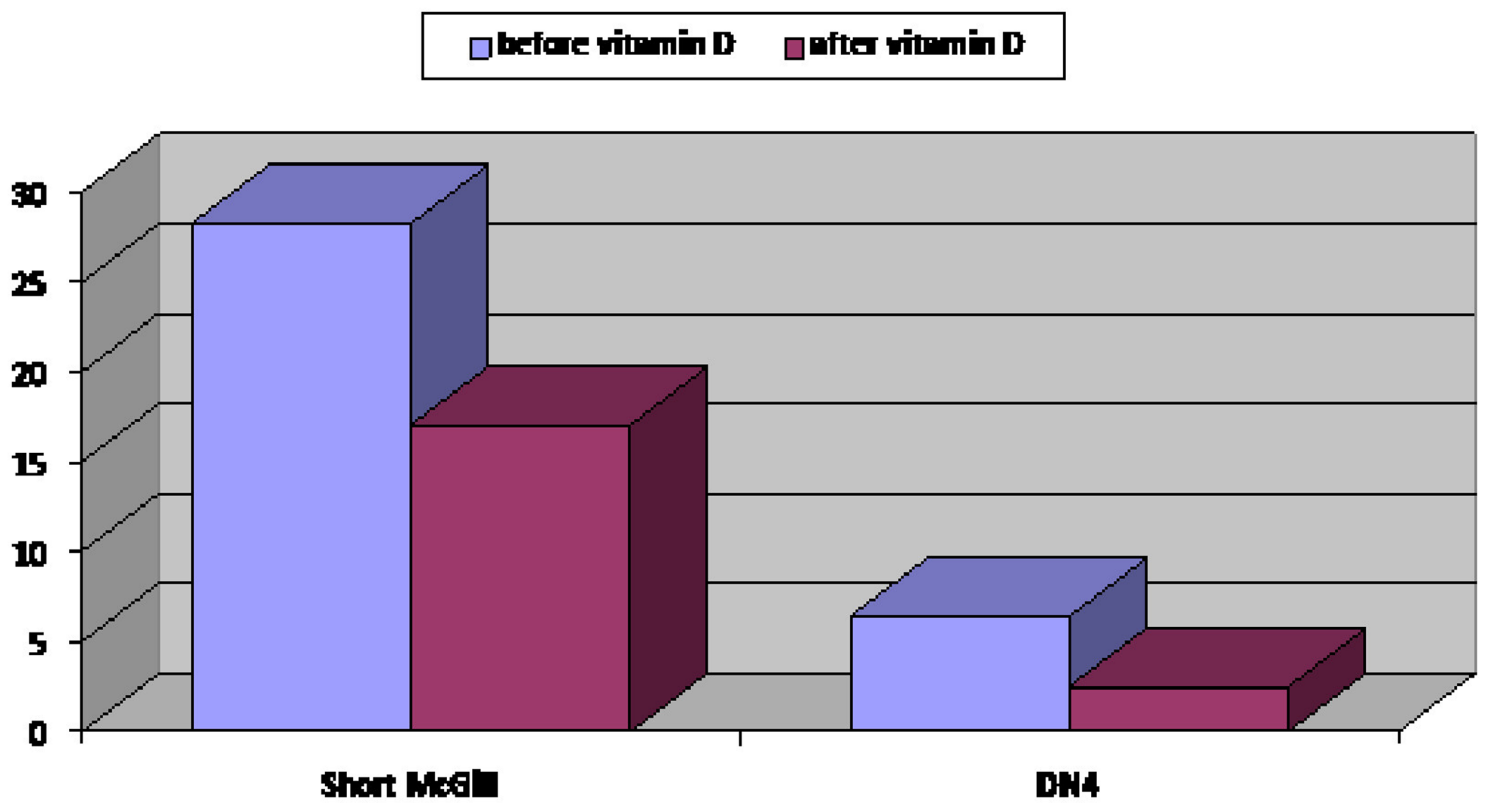
Comparison between neuropathic pain score assessed by Douleur Neuropathic 4 score and severity of peripheral neuropathy assessed by the Short-Form McGill Pain Questionnaire score of group (A) (prediabetic patients with peripheral neuropathy) before and after vitamin D supplementation.

We found a highly significant improvement in neuropathic pain severity as shown by the SF-MPQ (P≤0.001), there was also a significant reduction in the DN4 questionnaire score from (6.39 **±**1.64) to (2.5 **±**0.9) (≥4 denote neuropathic pain) with an improvement of neuropathic pain of about 82% and a total number of patients having a DN4 score less than 4 was 73 out of 89 prediabetic patients with peripheral neuropathy (P≤0.001) ( Figure 2). 

### Clinical examination for peripheral neuropathy

We found a highly significant improvement in vibration sense by tuning fork and protective sense measured by the 10 g monofilament test (P≤0.001), while there was no improvement regarding ankle reflex (P>0.05) ( Table 4).

**Table 4.** Comparison between Group (A) before and after vitamin D injection regarding clinical examination for peripheral neuropathy using ankle reflex, tuning fork (vibration) and 10 g monofilament.

	Group(A) before vitamin D		Group(A) after vitamin D		Sig. *
	Number	Percent	Number	Percent	P-value
**Ankle reflex**					
Absent	1	1.1%	1	1.1%	X²= 0.00 P= 1.00
Present	88	98.9%	88	98.9%	
Total	89	100.0%	89	100.0%	
**Vibration**					
Absent	43	48.3%	2	2.2%	X²= 52.8 **P** ≤ **0.001**
Reduced	11	12.4%	34	38.2%	
Present	35	39.3%	53	59.6%	
Total	89	100.0%	89	100.0%	
**Monofilament**					
Absent	68	76.4%	6	6.7%	X²=89.9 **P** ≤ **0.001**
Reduced	9	10.1%	38	42.7%	
Normal	12	13.5%	45	50.6%	
Total	89	100.0%	89	100.0%	

*Chi-square test between patients with peripheral neuropathy before and after vitamin D injection

## Discussion

Diabetic peripheral neuropathy in recently diagnosed diabetic patients may reach about 8% and more than 50% in patients with long-standing diabetes ^13^. Recently, the American Diabetes Association stated that there is no strong evidence that supports the lifestyle management or efficacy of glycemic control in the treatment of neuropathic pain, which means that pharmaceutical interventions such as pregabalin, duloxetine, or tapentadol are the only way of treatment ^14^. Accordingly, we aimed to demonstrate the association of vitamin D status with peripheral neuropathy and determine the effect of vitamin D supplementation on painful neuropathy in prediabetics.

Even with our sunny country, none of our patients (0%) had sufficient vitamin D level, while 85% (151 patients) were deficient and 15% (27 patients) were insufficient.

Kuchay *et al*., 2015 in their study found that prediabetes patients were 54.3% vitamin D deficient, 21.3% were insufficient and only 24.4% were sufficient despite abundant sunshine in India ^15^.

A negative correlation was found between serum 25 (OH) vitamin D and serum HbA1c, FBS and two hours post 75 g. Consistently, Kuchay *et al*., (2015) demonstrated an association between vitamin D status and prevalence of diabetes, with low prevalence in people with high vitamin D status and a belief that a serum 25(OH) vitamin D level of 15 ng/mL or less may be a threshold at which vitamin D deficiency confers negative effect on insulin sensitivity ^15^ This was confirmed when nearly 50% of patients with prediabetes had serum 25(OH) vitamin D levels below 15 ng/mL ^15^. On the contrary, Rolim *et al*., (2016) found the association between HbA1c and 25(OH) vitamin D controversial and glycemic control was not associated with vitamin D level ^16^. Luo *et al*., (2009) stated that there was no impact of hypovitaminosis D on metabolic syndrome status and HbA1c ^17^.

The association between vitamin D status and prevalence of diabetes can be explained through the effect of vitamin D on pancreatic β‐cell function and plasma calcium. Vitamin D deficiency decreases serum calcium, which regulates insulin synthesis and release ^18^.

On the other hand, administration of vitamin D causes increase in serum calcium, decrease in circulating free fatty acid levels, increase in insulin release and improvement in glucose levels ^19^.

Hypovitaminosis D in our patients was interestingly linked with the severity of peripheral neuropathy score elicited by the SF-MPQ scoring, DN4 questionnaire scoring and clinically by using 10 g monofilament, tuning fork and pinprick in nearly half of the patients, after adjusting for demographic data and other co-morbidities. Confirming these findings, Shehab *et al*., (2012) study on 210 diabetic patients, from which 87 had peripheral neuropathy, first found that vitamin D deficiency was significantly associated with diabetic peripheral neuropathy ^20^. In agreement with Shillo *et al*., (2019) who reported that serum vitamin D levels were lower in patients with painful DPN than in those with painless DPN, and pain scores were negatively correlated with serum vitamin D levels ^21^.

On the contrary *,* Basit *et al.,* (2016) acknowledged that there was no significant correlation between 25 (OH) vitamin D status with either total McGill pain location, McGill pain score, DN4 or positive symptoms ^22^. Studies by Usluogullari *et al*., (2015) also found no difference in the prevalence of vitamin D deficiency between diabetic peripheral neuropathy patients and controls ^23^.

Vitamin D level and two-hour post 75 g glucose were the independent predictors for neuropathy severity in our study, whereas Shehab *et al*., (2012) study confirmed that vitamin D was the only independent risk factor for diabetic peripheral neuropathy ^20^. While in China, He *et al*., (2017) declared that deficiency of vitamin D is an independent risk factor for diabetic peripheral neuropathy and can be considered a potential biomarker for peripheral neuropathy in diabetic Chinese patients ^24^.

On the other hand, Alkhatatbeh *et al*., (2019) showed that the only significant predictor for neuropathic pain was female gender, while vitamin D level, BMI, age, FBG, duration of T2DM, DBP and SBP were not ^25^. The divergence in the results of previous studies may be due to the use of different methods to assess neuropathy and because the studies were directed on different populations.

Injection of vitamin D 200.000 IU intramuscular every month for three successive months is in accordance with the guidelines for vitamin D supplementation and treatment of deficiency in Central Europe individuals with proved vitamin D deficiency which require higher doses of vitamin D than doses recommended for the general population. The therapeutic dose in severe deficiency should be 1.000–10.000 IU/day (~50.000 IU/week), depending on the patient’s body weight and age. The duration of the treatment varies from 1–3 months, depending on the degree of vitamin D deficiency ^26^. Our patients showed significant improvement and reduction in neuropathy severity score and also showed clinical improvement by monofilament and tuning fork. This is in line with Bell (2012) who found great improvement in neuropathic symptoms after supplementation with 50.000 IU of vitamin D _2_ every week in a case report of a patient suffering from diabetic peripheral neuropathy. The patient had been refractory to different types of treatment like tricyclic's, gabapentin, oxycodone and pregabalin ^27^. As well, Shehab *et al*., (2015) in their study applied vitamin D replacement therapy as a single intramuscular vitamin D dose of 300.000 IU and this application significantly enhanced the DN4 questionnaire scores of the patients with diabetic neuropathy ^28^. Correspondingly, Lee and Chen (2008) showed that oral cholecalciferol resulted in an approximate 50% reduction in painful neuropathic symptoms and a significant reduction in SF-MPQ score from 32.1 to 19.4; however, this study had neither a placebo group nor was randomized, leaving it open to considerable bias ^29^.

Possible explanation of previous studies was demonstrated *in vitro* by Fukuoka *et al*., (2001) and *in vivo* by Riaz *et al*., (1999) who considered vitamin D as a neurotrophic substance, which modulates neuronal growth and differentiation, and neuromuscular functions ^30, 31^. Its exact role in diabetic neuropathic pain is uncertain; insufficiency of vitamin D may increase damage of diabetic nerve and may affect the function of nociceptors leading to pain at a higher threshold of serum 25 (OH) vitamin D concentration higher than that in the non-diabetic individuals ^29^. 

Therefore, the results of previous studies corroborate our findings that vitamin D supplementation improves peripheral neuropathy and can be used as a safe treatment for peripheral neuropathy in prediabetic patients.

Opposing previous results, a study by Alam *et al*., (2016) reported no significant decrease in neuropathic pain scores after vitamin D administration ^32^. This study was based on all or none values instead of assessing the quantity of pain score, which may have led to a failure of observing a reduction in pain scoring.

Glycemic parameters of our patients showed significant improvement after the administration of 200.000 IU of vitamin D every four weeks for 12 weeks, which was the same result found by Kuchay *et al*., (2015) who revealed that correcting vitamin D deficiency in people with prediabetes significantly reduces FBG, two hours plasma glucose and A1C levels in 12 months ^15^. However, contrary to our findings, He *et al*., (2018) proclaimed in their meta-analysis that vitamin D supplementation did not improve fasting glucose levels or insulin resistance, nor did it prevent T2DM in non-diabetics ^33^. Furthermore, Moreira-Lucas *et al.,* (2017) confirmed that vitamin D supplementation did not improve fasting or post challenge measures of insulin sensitivity, β‐cell function or HbA1c ^34^. 

Among the limitations of the study were a small sample size compared to previous studies. Our study is the first to discuss the effect of vitamin D supplementation on peripheral neuropathy in prediabetic individuals whereas other studies have discussed the effect on diabetic patients. Finding prediabetic participants with peripheral neuropathy to include in the study was challenging.

## Conclusion

This study found that vitamin D deficiency can be considered an independent risk factor for peripheral neuropathy in prediabetic individuals. Also, correction of vitamin D deficiency improves glycemic parameters, lipid profile, peripheral neuropathy score and severity.

## Data availability

### Underlying data

Figshare: Underlying data for ‘The impact of vitamin D supplementation on peripheral neuropathy in a sample of Egyptian prediabetic individuals’, https://doi.org/10.6084/m9.figshare.15073287.v1 ^12^

This project contains the following underlying data:

- Data file 1: prediabetic patients without peripheral neuropathy and their descriptive and laboratory data.
- Data file 2: prediabetic with peripheral neuropathy and their descriptive, laboratory data, McGill Pain Questionnaire, clinical examination for neuropathy before vitamin D supplementation.
- Data file 3: prediabetic with peripheral neuropathy and their descriptive and laboratory data, McGill Pain Questionnaire, clinical examination for neuropathy after Vitamin D supplementation.

Data are available under the terms of the Creative Commons Zero “No rights reserved” data waiver (CC0 1.0 Public domain dedication).

## Consent statement

Written informed consent was obtained from all individual participants included in our study.
