# Supplementary material for: The impact of vitamin D supplementation on peripheral neuropathy in a sample of Egyptian prediabetic individuals
Source: F1000Res. 2021 Nov 1;10:817. Originally published 2021 Aug 16. [Version 2] doi: 10.12688/f1000research.55221.2 (PMC8851293; doi:10.12688/f1000research.55221.2)
Supplement: Peer review history

# Peer review: Reviewer response for version 2

**DOI:** 10.5256/f1000research.78627.r121333

**Published:** 2022-02-15

**Authors:** Ghada M. El Sagheer

**Affiliations:** 1 Department of Internal Medicine, Faculty of Medicine, Minia University, Minia, Egypt

Thanks to the authors for their choice of this subject of study which appears to be interesting and not frequently discussed before. It’s a really well-organized study, well-constructed title, discussing the effect of vitamin D supplementation on prediabetic individuals which is the first study so far discussing this specific point 

 The introduction was clear and updated, showing that microvascular complications like peripheral neuropathy can occur before the development of diabetes (Nebuchennykh M. *et al*., 2008 ^1^) and highlighting the association between peripheral neuropathy and vitamin D (Qu GB *et al*., 2017 ^2^).

 The methodology was perfectly planned, well written, and well-arranged so that the reader can understand the steps of the study and how the authors managed to find prediabetic individuals with peripheral neuropathy. Also, the number of patients is very suitable to the study design, helping to get more valid data on the effect of vitamin D supplementation on peripheral neuropathy as previous studies were lacking evidence due to either small sample size or defect in follow-up.

 The statistics are well prepared, the sample size is quite enough for the study and the data is analyzed by SPSS. The statistical analysis and tests are properly described and appropriate for the study, used correctly which helped to get the results that are interpreted in a clear way. The tables and figures are well presented and have a crucial role in the study and writing of the manuscript and helped to finalize the results of the study. Finally, data files are uploaded which will help produce more results further on it, but it would be much better to present the raw data in a simplified way.

 The results were nicely presented; the authors first presented that vitamin D deficiency was highly prevalent in the studied group. Second, the authors compared clinical and laboratory data between the two studied groups. Third, they presented the effect of vitamin D supplementation over the arm with the peripheral neuropathy and clarified the benefit on the vitamin D levels and neuropathy score and severity and clinically by peripheral neuropathy indicators (ankle reflex, tuning fork vibration, and 10 g monofilament).

 The discussion was organized and updated, citing each part of the study with other papers, discussing with cons, and clarifying the cause of each result.

 The conclusion was short, to the point, summarizing all the aims in the study design.

 Despite this, I would be thankful if the authors could clarify the following points:

1. The main results are not sufficiently presented in the abstract (e.g. the mean and SD of the vitamin D level in the study groups, the mean of HbA1c results, and the other glucose parameters, etc). 
2. In the methods section, I need to know whether the pre-diabetic individuals received any management, e.g. lifestyle modifications, exercise, metformin, to clarify that neuropathy improvement is not related to other factors.
3. What are the definitions of vitamin D deficiency, insufficiency, and sufficiency the authors used, and which reference? 
4. In the results sections, the mean level of vitamin D of the studied groups was not written in any part of the results or tables.

Finally, I find this study accepted, well-organized, and well-written.

Is the work clearly and accurately presented and does it cite the current literature?

Yes

If applicable, is the statistical analysis and its interpretation appropriate?

Yes

Are all the source data underlying the results available to ensure full reproducibility?

Yes

Is the study design appropriate and is the work technically sound?

Yes

Are the conclusions drawn adequately supported by the results?

Yes

Are sufficient details of methods and analysis provided to allow replication by others?

Yes

Reviewer Expertise:

Endocrine disorders

I confirm that I have read this submission and believe that I have an appropriate level of expertise to confirm that it is of an acceptable scientific standard.

## References

[1] : Idiopathic polyneuropathy and impaired glucose metabolism in a Norwegian patient series. *Eur J Neurol* .2008;15(8) : 10.1111/j.1468-1331.2008.02197.x 810-6 10.1111/j.1468-1331.2008.02197.x 18549398

[2] : The association between vitamin D level and diabetic peripheral neuropathy in patients with type 2 diabetes mellitus: An update systematic review and meta-analysis. *J Clin Transl Endocrinol* .2017;9: 10.1016/j.jcte.2017.04.001 25-31 10.1016/j.jcte.2017.04.001 29067266PMC5651294

# Peer review: Reviewer response for version 2

**DOI:** 10.5256/f1000research.78627.r101602

**Published:** 2022-01-24

**Authors:** Zaynab Alourfi, Ibrahim Alali

**Affiliations:** 1 Faculty of Medicine, Damascus University, Damascus, Syria; 2 Damascus University, Damascus, Syria

This article discusses a common health problem facing diabetologists and internal medicine professionals.

- The authors have properly introduced the aim of the study in the introduction, especially the fourth paragraph. For instance, a lot of papers (Xiaohua G *et al*., 2021 ^1^ and Shehab D *et al*., 2012 ^2^) talked about Vit D deficiency in relation to diabetic neuropathy, and in clinical practice a lot of Vit D prescriptions are without clear evidence, but above that, no data about prediabetes, so focusing on this point is great.
- The methodology was clear after the latest revision and helps to reproduce results by other researchers.
- I prefer that the 75 g glucose tolerance test be referred to with “75g-OGTT” rather than “2h-PPBG” in the laboratory studies and other places.
- Regarding statistical analysis, the authors described statistical tests properly but to give an accurate criticism, the reviewer should be more specialized in statistics. Data files are uploaded completely and allow who wants to use them in reproducing results.
- Results were well described and organized; authors started with sample characteristics and organized them in Table 1 properly, then moved to Vit D results and followed up with results after replacement, then described neuropathic pain scores and also organized it in tables.
- The discussion was very good, citing the literature and comparing results to other papers was well established, and I really appreciate the authors' comment about how hard it is to find patients with such inclusion criteria given my expertise. The authors declared that one limitation was collecting patients compatible with the inclusion criteria, it's really challenging to find a prediabetic patient accept, confirming the diagnosis by fasting and doing OGTT test then assessment of neuropathic pain, so this effort from the authors is really appreciated in my opinion.
- The conclusion was short but I think it is enough to support the results since it is mentioning the primary outcome for this paper which is that replacement of Vit D in neuropathic prediabetes patients is useful.

Finally, I find this version accepted and well written.

Is the work clearly and accurately presented and does it cite the current literature?

Yes

If applicable, is the statistical analysis and its interpretation appropriate?

I cannot comment. A qualified statistician is required.

Are all the source data underlying the results available to ensure full reproducibility?

Yes

Is the study design appropriate and is the work technically sound?

Yes

Are the conclusions drawn adequately supported by the results?

Yes

Are sufficient details of methods and analysis provided to allow replication by others?

Yes

Reviewer Expertise:

Vitamin D disorders, osteoporosis, hypothyroidism, diabetes

We confirm that we have read this submission and believe that we have an appropriate level of expertise to confirm that it is of an acceptable scientific standard.

## References

[1] : Severe Vitamin D Deficiency Is Associated With Increased Expression of Inflammatory Cytokines in Painful Diabetic Peripheral Neuropathy. *Front Nutr* .2021;8: 10.3389/fnut.2021.612068 612068 10.3389/fnut.2021.612068 33777989PMC7987807

[2] : Does Vitamin D deficiency play a role in peripheral neuropathy in Type 2 diabetes?. *Diabet Med* .2012;29(1) : 10.1111/j.1464-5491.2011.03510.x 43-9 10.1111/j.1464-5491.2011.03510.x 22050401
